# Proteomics signature of autoimmune atrophic gastritis: towards a link with gastric cancer

**DOI:** 10.1007/s10120-020-01148-3

**Published:** 2021-02-23

**Authors:** Ombretta Repetto, Valli De Re, Paolo Giuffrida, Marco Vincenzo Lenti, Raffaella Magris, Marino Venerito, Agostino Steffan, Antonio Di Sabatino, Renato Cannizzaro

**Affiliations:** 1 Facility of Bio-Proteomics, Immunopathology and Cancer Biomarkers, Centro di Riferimento Oncologico di Aviano (CRO) IRCCS, Aviano, PN Italy; 2 Department of Internal Medicine, San Matteo Hospital Foundation, University of Pavia, Pavia, Italy; 3 Gastroenterology, Centro di Riferimento Oncologico di Aviano (CRO) IRCCS, Aviano, PN Italy; 4 Department of Gastroenterology, Hepatology and Infectious Diseases, Otto-von-Guericke University Hospital, Magdeburg, Germany; 5 Immunopathology and Cancer Biomarkers, Centro di Riferimento Oncologico di Aviano (CRO) IRCCS, Aviano, PN Italy

**Keywords:** Autoimmune disease, Biological markers, Gastric cancer, Gastritis, Proteomics

## Abstract

**Background:**

Autoimmune atrophic gastritis (AAG) is a chronic disease that can progress to gastric cancer (GC). To better understand AAG pathology, this proteomics study investigated gastric proteins whose expression levels are altered in this disease and also in GC.

**Methods:**

Using two-dimensional difference gel electrophoresis (2D-DIGE), we compared protein maps of gastric corpus biopsies from AAG patients and controls. Differentially abundant spots (|fold change|≥ 1.5, *P* < 0.01) were selected and identified by LC–MS/MS. The spots were further assessed in gastric antrum biopsies from AAG patients (without and with *Helicobacter pylori* infection) and from GC patients and unaffected first-degree relatives of GC patients.

**Results:**

2D-DIGE identified 67 differentially abundant spots, with 28 more and 39 less abundant in AAG-corpus than controls. LC–MS/MS identified these as 53 distinct proteins. The most significant (adjusted *P* < 0.01) biological process associated with the less abundant proteins was “tricarboxylic acid cycle”. Of the 67 spots, 57 were similarly differentially abundant in AAG-antrum biopsies irrespective of *H. pylori* infection status. The differential abundance was also observed in GC biopsies for 14 of 28 more abundant and 35 of 39 less abundant spots, and in normal gastric biopsies of relatives of GC patients for 6 and 25 spots, respectively. Immunoblotting confirmed the different expression levels of two more abundant proteins (*PDIA3, GSTP* gene products) and four less abundant proteins (*ATP5F1A, PGA3, SDHB, PGC*).

**Conclusion:**

This study identified a proteomics signature of AAG. Many differential proteins were shared by GC and may be involved in the progression of AAG to GC.

**Supplementary Information:**

The online version contains supplementary material available at 10.1007/s10120-020-01148-3.

## Introduction

Autoimmune atrophic gastritis (AAG) is a T-cell-driven disease characterized by inflammation, loss of the oxyntic mucosa of the gastric corpus and fundus, and ultimately complete atrophy of the stomach mucosa [1, 2]. Patients with AAG have autoantibodies against H + K + ATPase, a parietal cell protein [3]. Antibody-mediated destruction of parietal cells reduces the production of intrinsic factor [4] and leads to the progressive loss of acid secretion, resulting in hypochlorhydria, hypergastrinemia, and hyperplasia of both G cells [5] and enterochromaffin-like cells [6]. Oxyntic atrophy leads to a loss of zymogenic chief cells, reducing pepsinogen I release [7]. AAG is diagnosed by endoscopy and serological detection of autoantibodies against parietal cells [8] and intrinsic factor [9]. Serological testing for gastrin-17 and pepsinogen I and II helps in the diagnosis: high gastrin-17 levels or a low pepsinogen I/II ratio suggests AAG [10, 11].

AAG is also known as type A gastritis. It is distinguished from type B gastritis, which is caused by *Helicobacter pylori* infection, environmental factors or diet, and which has an antrum-predominant anatomic distribution [12, 13]. However, many patients with AAG of the gastric corpus have *H. pylori* infection [14]. In a late stage of atrophy, inflammation decreases, the oxyntic mucosa is replaced by metaplastic epithelium, and the hyperplasia of enterochromaffin-like cells may progress to type I gastric carcinoid or adenocarcinoma [15–18]. Gastric atrophy and intestinal metaplasia are preneoplastic conditions. Patients with severe atrophy and intestinal metaplasia have an increased risk of intestinal-type gastric cancer (GC), and the risk increases with lesion severity [17, 19, 20].

Little is known about AAG pathogenesis because: (i) the disease has low prevalence, (ii) patients may have concomitant *H. pylori* infection, (iii) the disease causes few symptoms in early stages, and (iv) it is detectable only by endoscopy [21, 22]. Therefore, molecular characterization of AAG tissue should provide insight into the pathogenesis of this disease and into the process by which it progresses to neoplasia. One approach now widely used to molecularly characterize tissue specimens is provided by proteomics. In particular, the method called difference gel electrophoresis (DIGE) has been successfully used to characterize many human cancers (reviewed in Ref. [23]), proving useful in the discovery of tissue markers for GC [24], colorectal cancer [25], ulcerative colitis and Crohn's disease [26], but not yet for AAG.

We used DIGE in a quantitative proteomics study of gastric corpus biopsies from patients with AAG and patients in whom AAG was excluded, allowing us to identify a set of 53 differentially abundant proteins (67 spots). To test the specificity of these findings, we examined the abundance of these proteins in additional biopsies, including from the antrum of AAG patients, without or with *H. pylori* infection. Moreover, to shed light on the neoplastic progression of AAG, we examined their abundance in biopsies from GC patients and from first-degree relatives of GC patients, who are at risk of GC development [27].

## Methods

### Study design

The study enrolled consecutive patients undergoing esophagogastroduodenoscopy and gastric biopsy at the Unit of Oncological Gastroenterology of Centro di Riferimento Oncologico di Aviano (CRO) and Fondazione IRCCS San Matteo Hospital between April 2014 and December 2016. Patients whose tissues were analyzed gave written informed consent. The study was approved by CRO Institutional Review Board (decision no. 14) and San Matteo Hospital Institutional Ethics Committee (protocol no. 20170000832).

The study was organized in three parts. In Part I, gastric corpus biopsy specimens were examined from nine patients subsequently diagnosed with AAG (called “AAG-corpus”) and from nine patients in whom gastric disease was histologically excluded (controls); these samples had been collected at the first endoscopy examination, at San Matteo Hospital. Proteins that were differentially abundant between the groups were identified. In Part II, these differentially abundant proteins were examined in additional sets of biopsy specimens from patients recruited at CRO: (1) nine gastric antrum biopsies from AAG patients (AAG-antrum) without *H. pylori* infection; (2) nine gastric antrum biopsies from AAG patients with *H. pylori* infection (AAG-antrum-HP); (3) six gastric biopsies from first-degree relatives of GC patients (FDR-GC); and (4) six tumor biopsies from GC patients. The differential abundance of the proteins in these biopsy sets was compared to the controls of Part I. In Part III, validation analyses for seven spots were done by: (i) examining additional FDR-GC and GC proteome maps (six each), and (ii) immunoblotting pooled samples from Parts I and II.

### Diagnostic procedures

All study subjects underwent esophagogastroduodenoscopy with gastric sampling according to the updated Sydney system [28]. At least two biopsies from the antrum, one from the incisura angularis, two from the corpus and two from each visible lesion were taken.

According to international criteria [1, 28], AAG was diagnosed when any grade of atrophy of the oxyntic mucosa was present, sparing the antrum. A diagnosis of AAG was excluded in subjects (controls) who had esophagogastroduodenoscopy for dyspepsia or other upper gastrointestinal complaints, when the examination and histopathological assessment were unremarkable and antiparietal cell antibodies were absent. Gastric cancer was diagnosed histologically based on Lauren’s criteria [29]. First-degree relatives of GC patients underwent esophagogastroduodenoscopy and biopsy for GC surveillance, and their gastric tissue was included when free of neoplasia and other gastric abnormalities.

AAG was also diagnosed serologically according to the presence of antiparietal cell antibodies [30]. Antibody levels were estimated by indirect immunofluorescence using a kit from Euroimmun (Lübeck, Germany). *H. pylori* infection was diagnosed on tissue sections using hematoxylin and eosin and Giemsa stain.

### Sample preparation

Biopsy specimens were transported at 4 °C and stored at − 80 °C. Soluble proteins were extracted from frozen biopsies. Briefly, each frozen sample was homogenized in 200 µL 30 mM Tris HCl pH 8.5, 4% (w/v) CHAPS, 7 M urea, 2 M thiourea, and protease inhibitor cocktail (cOmplete EDTA-free tablets; Roche) on ice using Sample Grinding Kits (Cytiva—formerly GE Healthcare Life Sciences). The samples were lysed (4 °C, 15 min) and then centrifuged (12,000 *g*, 4 °C, 5 min). Supernatants were treated with 2-D Clean-Up Kit (Cytiva) and centrifuged. The resulting pellets were resuspended in 60 µL rehydration buffer (7 M urea, 2 M thiourea, 4% (w/v) CHAPS). Protein concentration was determined with Bradford reagent (Bio-Rad). Samples were stored at − 80 °C until 2D-DIGE.

### 2D-DIGE and image analysis

For 2D-DIGE, an internal standard was prepared by pooling equal amounts of protein extracts including all samples used in this study. This standard was labeled with Cy2 dye while individual samples were labeled, in equal numbers, with either Cy3 or Cy5 (CyDye DIGE Fluor Minimal Labeling Kit; Cytiva). Then, internal standard, one Cy3-labeled sample and one Cy5-labeled sample (25 µg each) were combined and separated by isoelectrofocusing (IEF) on Immobiline Drystrip gels with a nonlinear pH gradient (pH 3–10 NL IPG, Cytiva) for 30 kVh on a Protean IEF Cell system (Bio-Rad). After IEF, strips were frozen at − 80 °C. For the second dimension, strips were defrosted, incubated 15 min in equilibrating buffer (4 M urea, 2 M thiourea, 50 mM Tris HCl pH 8.9, 30% glycerol, 2% SDS) containing 65 mM DTT and 15 min in equilibrating buffer containing 135 mM iodoacetamide, and subjected to electrophoresis on 8–16% polyacrylamide gels (Criterion TGX, Bio-Rad) in running buffer (25 mM Tris pH 8.3, 192 mM glycine, 0.1% SDS) at 120 V for 20 min and 150 V for 50 min.

Gel images were acquired using an Amersham Typhoon Scanner (Cytiva) at 100 µm. Images were analyzed with DeCyder image analysis software v6.5 (Cytiva). The DyCyder Differential In-gel Analysis module detected spots in each gel and normalized their staining intensity to the same spot of the internal standard (standardized abundance). The Biological Variation Analysis module compared the standardized abundance of spots across gels and calculated an average ratio (fold change) between the experimental and control groups. Differentially abundant spots were those that: (i) were present on > 70% of spot maps per group; (ii) had a standardized abundance average ratio (fold change) of ≥ 1.50 or ≤ − 1.50; and (iii) had *P* < 0.01 on Student’s *t* test. The differentially abundant spots were then analyzed using the Extended Data Analysis module for principal component analysis and hierarchical clustering. Multiple group comparison of standardized spot abundance was performed with one-way ANOVA with a significance level of *P* < 0.01.

### Protein identification by MS

To identify the proteins of the differentially abundant spots, a 2D-DIGE pick gel was prepared and separated. After Coomassie blue staining, spots of interest were excised with the Screen Picker (Proteomics Consult), destained overnight with 25 mM ammonium bicarbonate in 50% acetonitrile, dehydrated with 100% acetonitrile, dried, and digested overnight with trypsin (200 ng/spot; T6567 Sigma-Aldrich) in 40 mM ammonium bicarbonate, 9% acetonitrile at 37 °C. The trypsin digest was extracted with 1% trifluoroacetic acid (Sigma-Aldrich), lyophilized and stored at − 80 °C until shipping.

Samples were analyzed at the Proteomics Facility of CEINGE-Biotecnologie Avanzate (Naples, Italy). LC–MS/MS was done on a Proxeon EASY nano liquid chromatography system coupled with an LTQ Orbitrap XL mass spectrometer with ETD (Thermo Fisher Scientific, Massachusetts, USA). CEINGE provided raw MS data in.mgf format.

Mascot Server v2.3 (Matrix Science, Boston, USA) was used to search for matches between the MS data and proteins in the NCBI nr and Swiss-Prot databases, selected for human taxonomy.

### Protein functional annotation

Functional annotation of the differentially abundant proteins between AAG-corpus and control samples was done with DAVID 6.8 [31]. Gene ontology (GO) biological processes, molecular functions and cellular components associated with the proteins were reported together with *P *values (Fisher’s exact test) and adjusted *P *values (Benjamini–Hochberg procedure). Strongly enriched annotation categories (adjusted *P* < 0.05) were considered.

### Immunoblotting

Differentially abundant proteins of interest were examined by immunoblotting of pools of protein extracts from all experimental groups and controls from Parts I and II. Protein (10 µg per pool) was fractionated on 12% Criterion TGX Stain-Free gels (Bio-Rad). Gel images were acquired with the Chemidoc system (Bio-Rad) to document equal protein loading among samples. Then, proteins were electrotransferred onto nitrocellulose membranes and probed with following primary antibodies: anti-ATP5F1A (1:1000; #ab151229, AbCam), anti-SDHB [21A11AE7] (1:500; #ab14714, AbCam), anti-PGA3 (1:1000; #PA5-49728, Thermo Fisher Scientific), anti-pepsinogen II/PGC (1:500; #ab135862, AbCam), anti-PDIA3 (internal region; 1:1000; #ABIN3187755, Antibodies-online; Aachen, Germany), anti-GSTP1 (1:1000; #PA5-29558, Thermo Fisher Scientific), and anti-PSME1 (1:1000; #ab14714, AbCam). Primary antibodies were detected with HRP-conjugated goat anti-rabbit and anti-mouse IgG Fc fragment antibodies (1:10,000 dilution; Bethyl Laboratories, Montgomery (TX), USA) and Clarity Western ECL Substrate (Bio-Rad). Blots were imaged with the Chemidoc system.

## Results

### Differentially abundant proteins in AAG

In Part I of this study, protein abundance in the gastric corpus was compared between nine AAG patients (AAG-corpus) and nine controls without AAG (Table 1). Six of nine AAG patients had intestinal metaplasia. No study subject had *H. pylori* infection, by study design*.*

**Table 1:** Clinicopathological characteristics of the study groups, by study design

Group	Gastric biopsy (*n*)	Cases (*n*)	Female (*n*)	*H. pylori* infection (*n*)	Age (years)^a^
Part I					
AAG-corpus^b^	Corpus (9)	9	6	0	51 (7)
Controls	Corpus (9)	9	3	0	47 (13)
Part II					
AAG-antrum^b^	Antrum (9)	9	6	0	50 (14)
AAG-antrum-HP^b^	Antrum (9)	9	5	9	45 (11)
FDR-GC	Antrum (5), corpus (1)	6	4	0^d^	44 (6)
GC^c^	Antrum (1), corpus (4), fundus (1)	6	3	0^d^	63 (14)
Part III					
FDR-GC	Antrum (6)	6	4	0^d^	62 (12)
GC^c^	Antrum (3), corpus (3)	6	2	4^d^	64 (13)

*AAG* autoimmune atrophic gastritis, *FDR-GC* first-degree relative of a patient with gastric cancer, *GC* gastric cancer, HP *H. pylori* infection, *ND* not determined

^a^Age at biopsy, mean (SD)

^b^Six patients had intestinal metaplasia

^c^Lauren’s histological classification: intestinal type (*n* = 1), diffuse type (*n* = 2), indeterminate (*n* = 2); data missing for one case

^d^Data missing for two cases

**Fig. 1:**
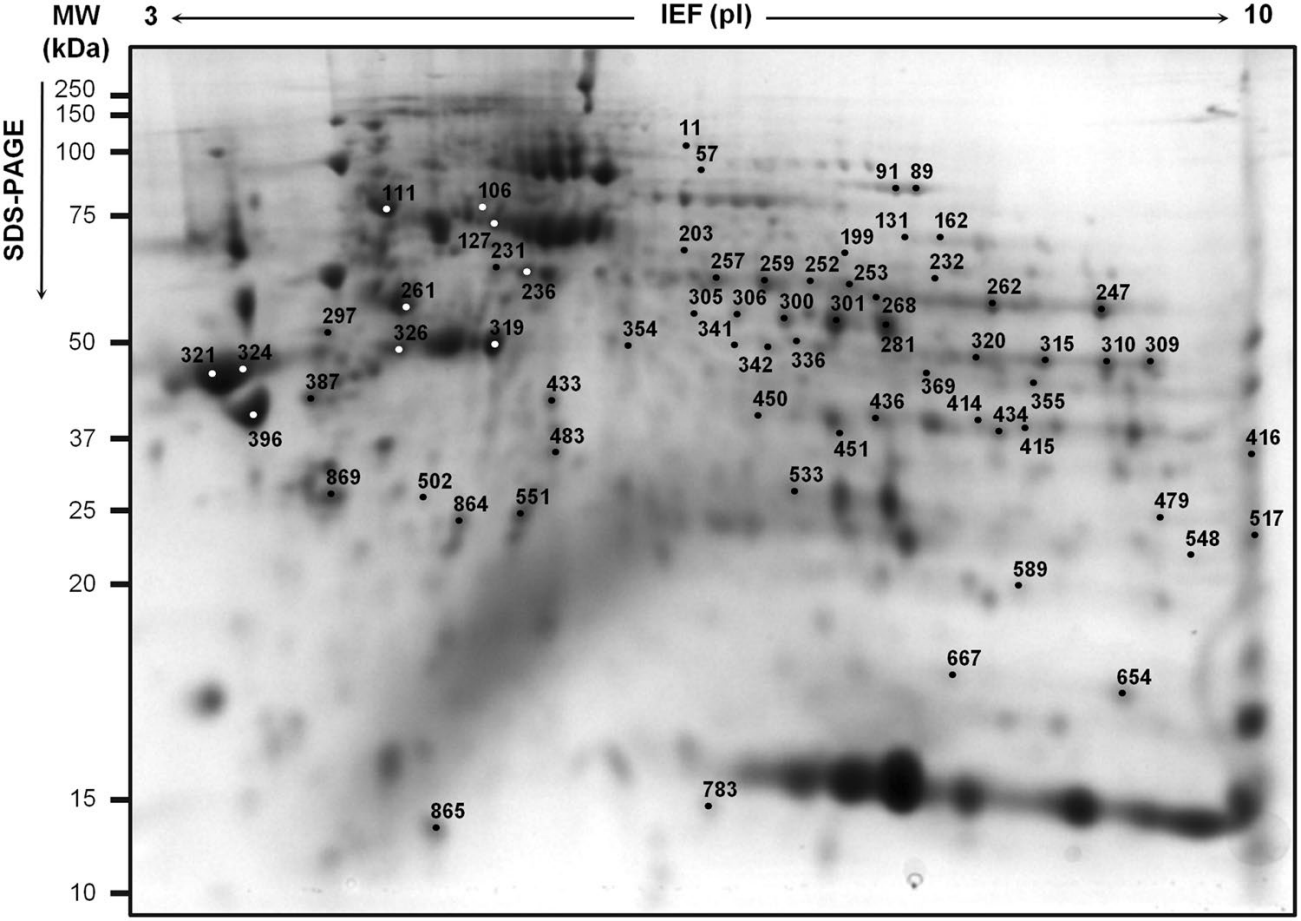
Two-dimensional proteome map of pooled proteins (450 µg) of gastric corpus biopsy specimens from patients with AAG-corpus and controls. Proteins were resolved on an immobilized pH 3–10 gradient, followed by SDS-PAGE (8–16% acrylamide). The numbers indicate the 67 differentially abundant spots between AAG-corpus and control groups (i.e. present on > 70% of spot maps, |fold change|≥ 1.5, Student's *t* test *P* < 0.01). IEF, isoelectric focusing

**Fig. 2:**
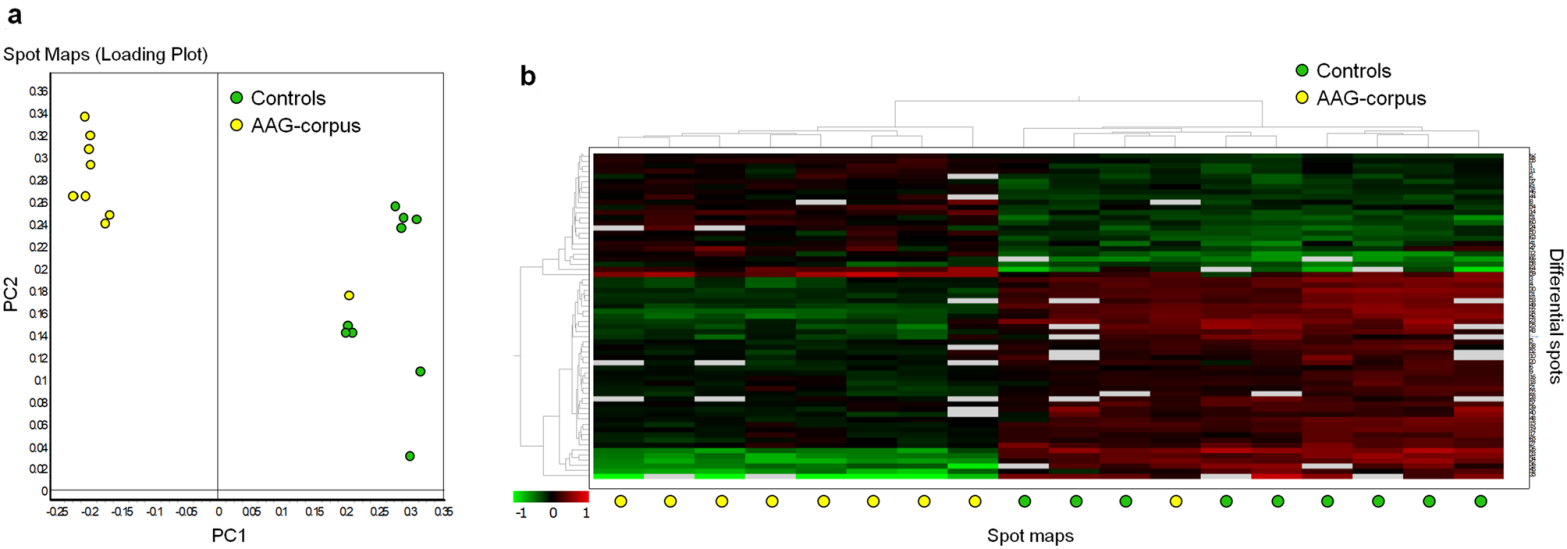
Grouping of differentially abundant spots on 2D-DIGE between AAG-corpus and control groups.** a** Principal component analysis. Each circle represents an individual spot map.** b** Hierarchical clustering. The dendrogram on the left orders the spots so that similar data are displayed next to each other. The dendrogram on the top orders the samples by similarity

Each gastric biopsy was processed by 2D-DIGE to generate a proteome map (Fig. 1). Comparison of proteome maps between the two groups identified 67 spots as differentially abundant (*P* < 0.01, Student’s *t* test). In particular, 28 spots were more abundant in the AAG-corpus group and 39 spots were less abundant, compared to controls. At principal component analysis of the 67 spots, the nine spot maps in each group grouped together with the exception of one AAG-corpus sample that grouped with controls (Fig. 2a). Similar results were obtained with hierarchical clustering (Fig. 2b). No particular clinical or histological findings explained the misclassification of the one AAG-corpus sample.

The 67 differentially abundant spots were identified by LC–MS/MS as 53 distinct proteins (Table 2). In particular, 25 proteins were more abundant in the AAG-corpus group, with a fold change from 1.5 to 4.4 with respect to controls. Furthermore, 28 proteins were less abundant, compared to controls, with a fold change from − 1.5 to − 13.0.

**Table 2:** Differentially abundant proteins in gastric corpus tissue between patients with autoimmune atrophic gastritis (AAG) and controls

Spot no.^a^	MW (pI)^b^	Database	Accession	Gene	Protein	Score	Matches	Seq	Seq. cov. %	Fold change^c^	*P *value
More abundant in AAG (*n* = 28)											
783*	16,102 (6.8)	SwissProt	HBB_HUMAN	HBB	Hemoglobin subunit beta	177	8	6	42	4.4	1.71E–04
865*°	10,161 (5.3)	NCBInr	gi|20,664,042	S100A6	Chain A, crystal structure of calcium-free (or Apo) human S100a6	155	22	5	32	3.3	1.64E–06
	11,826 (5.2)	SwissProt	S10AE_HUMAN	S100A14	Protein S100-A14	103	3	3	33	–	–
231*	54,541 (5.6)	NCBInr	gi|220,702,506	PDIA3^d^	Chain A, protein disulfide-isomerase A3 erp57	808	50	33	54	2.6	6.41E–04
236	54,541 (5.6)	NCBInr	gi|220,702,506	PDIA3^d^	Chain A, protein disulfide-isomerase A3 erp57	1380	93	39	56	2.4	8.16E-05
551*°	23,583 (5.4)	NCBInr	gi|726,098	GSTP1-1	Glutathione S-transferase P1-1 class	7083	261	12	64	2.2	3.55E–06
436°	38,808 (7.6)	SwissProt	ANXA2_HUMAN	ANXA2	Annexin A2	673	56	27	57	2.1	3.75E–04
297	53,676 (5.1)	SwissProt	VIME_HUMAN	VIM	Vimentin	1255	79	34	53	2.1	4.22E-03
548	22,653 (8.9)	SwissProt	TAGL_HUMAN	TAGLN2	Transgelin	404	28	16	74	2.1	5.14E-03
415	38,808 (7.6)	SwissProt	ANXA2_HUMAN	ANXA2	Annexin A2	522	37	25	56	2.0	7.37E–03
434*°	38,808 (7.6)	SwissProt	ANXA2_HUMAN	ANXA2	Annexin A2	912	77	27	58	1.9	2.09E-05
667*	18,719 (8.2)	SwissProt	COF1_HUMAN	CFL1	Cofilin-1	151	23	11	39	1.8	1.09–03
354	42,829 (5.9)	SwissProt	ILEU_HUMAN	SERPINB1	Leukocyte elastase inhibitor	635	33	22	48	1.8	3.49E–03
131*	61,305 (7.3)	NCBInr	gi|237,823,914	FGA	Chain A, crystal Structure of human fibrinogen	479	29	20	37	1.8	2.94E–03
479*°	26,193 (8.6)	SwissProt	LEG3_HUMAN	LGALS3	Galectin-3	712	53	11	36	1.6	1.30E–03
57	74,380 (6.6)	SwissProt	LMNA_HUMAN	LMNA	Prelamin-A/C	621	42	32	37	1.7	1.32E–03
	96,590 (6.1)	SwissProt	PDC6I_HUMAN	PDCD6IP	Programmed cell death 6-interacting protein	616	47	33	29	–	–
387	33,027 (4.6)	NCBInr	gi|47,519,616	TPM2	Tropomyosin beta chain isoform 2	1171	93	39	78	1.7	9.30E–04
326*	44,079 (5.0)	SwissProt	K1C19_HUMAN	KRT19	Keratin, type I cytoskeletal 19	4978	280	38	73	1.7	1.86E–04
589*	22,324 (8.3)	SwissProt	PRDX1_HUMAN	PRDX1	Peroxiredoxin-1	807	81	15	56	1.6	6.91E–06
654*	18,719 (8.2)	SwissProt	COF1_HUMAN	CFL1	Cofilin-1	643	80	14	45	1.6	2.65E–04
232*	51,926 (8.1)	NCBInr	gi|5,453,595	CAP1	Adenylyl cyclase-associated protein	984	46	19	45	1.5	4.34E-04
11	74,380 (6.6)	SwissProt	LMNA_HUMAN	LMNA	Prelamin-A/C	657	39	31	35	1.5	6.52E–03
	117,059 (6.4)	SwissProt	ODO1_HUMAN	OGDH	2-oxoglutarate dehydrogenase, mitochondrial	479	45	28	22	–	–
533*°	28,909 (6.6)	SwissProt	CAH1_HUMAN	CA1	Carbonic anhydrase 1	700	50	11	54	1.5	9.17E–03
203	61,066 (6.1)	SwissProt	TCPG_HUMAN	CCT3	T-complex protein 1 subunit gamma	917	54	29	43	1.5	7.36E–03
869	27,899 (4.7)	SwissProt	1433Z_HUMAN	YWHAZ	14–3-3 protein zeta/delta (protein kinase C inhibitor protein 1)	1472	173	23	60	1.5	4.31E–06
483	28,876 (5.8)	SwissProt	PSME1_HUMAN	PSME1	Proteasome activator complex subunit 1	961	95	53	58	1.5	1.41E–03
127*	70,294 (5.5)	SwissProt	HS71A_HUMAN	HSPA1A	Heat shock 70 kDa protein 1A	1441	94	38	46	1.5	9.55E–03
414	38,808 (7.6)	SwissProt	ANXA2_HUMAN	ANXA2	Annexin A2	592	36	24	56	1.5	1.23E–04
502	23,031 (5.1)	SwissProt	GDIR2_HUMAN	ARHGDIB	Rho GDP-dissociation inhibitor 2	599	26	9	40	1.5	5.53E–05
Less abundant in AAG (*n* = 39)											
321*°	42,349 (4.2)	SwissProt	PEPA3_HUMAN	PGA3	Pepsinogen-3 (pepsin A-3)	713	112	9	11	− 13.0	4.9E–05
301*°	45,380 (6.8)	SwissProt	LIPG_HUMAN	LIPF	Gastric triacylglycerol lipase	3625	154	10	22	− 5.3	4.2E–06
319*°	42,617 (5.3)	SwissProt	KCRB_HUMAN	CKB	Creatine kinase B-type	11,761	455	23	56	− 4.8	4.5E–06
396*	42,798 (4.4)	SwissProt	PEPC_HUMAN	PGC	Gastricsin (pepsinogen C)	2384	64	4	8	− 4.5	2.6E–05
324*°	42,349 (4.2)	SwissProt	PEPA3_HUMAN	PGA3	Pepsinogen-3 (pepsin A-3)	168	66	10	12	− 4.1	3.1E–04
320*°	51,333 (8.9)	SwissProt	IDHP_HUMAN	IDH2	Isocitrate dehydrogenase, mitochondrial	1220	75	24	40	− 4.1	2.2E–05
315*°	51,333 (8.9)	SwissProt	IDHP_HUMAN	IDH2	Isocitrate dehydrogenase, mitochondrial	1592	111	26	45	− 3.4	2.1E–05
355*°	45,456 (9.0)	SwissProt	THIL_HUMAN	ACAT1	Acetyl-CoA acetyltransferase, mitochondrial	784	41	23	44	− 3.1	5.8E–05
300*	45,380 (6.8)	SwissProt	LIPG_HUMAN	LIPF	Gastric triacylglycerol lipase	531	57	7	15	− 3.1	6.0E–04
433*°	36,900 (5.7)	SwissProt	LDHB_HUMAN	LDHB	L-lactate dehydrogenase B chain	1772	145	21	45	− 3.0	6.3E–06
89*°	86,113 (7.4)	SwissProt	ACON_HUMAN	ACO2	Aconitate hydratase, mitochondrial	912	57	27	31	− 2.9	9.0E–06
281*°	45,380 (6.8)	SwissProt	LIPG_HUMAN	LIPF	Gastric triacylglycerol lipase	1492	82	9	22	− 2.9	2.0E–05
309*°	51,333 (8.9)	SwissProt	IDHP_HUMAN	IDH2	Isocitrate dehydrogenase	1082	78	25	42	− 2.8	1.9E-05
310*°	51,333 (8.9)	SwissProt	IDHP_HUMAN	IDH2	Isocitrate dehydrogenase	1527	112	24	39	− 2.6	1.5E–05
91*°	86,113 (7.4)	SwissProt	ACON_HUMAN	ACO2	Aconitate hydratase, mitochondrial	827	52	30	33	− 2.5	2.9E–05
306*°	47,481 (7.01)	SwissProt	ENOA_HUMAN	ENO1	Alpha-enolase	386	32	19	35	− 2.3	5.6E–03
451*	37,571 (6.50)	NCBInr	gi|4,104,867	AFAR	Aflatoxin B1-aldehyde reductase	985	48	14	36	− 2.3	2.3E–04
341*°	49,852 (7.26)	SwissProt	EFTU_HUMAN	TUFM	Elongation factor Tu, mitochondrial	145	7	7	13	− 2.2	4.3E–04
369*	45,456 (9.0)	SwissProt	THIL_HUMAN	ACAT1	Acetyl-CoA acetyltransferase, mitochondrial	822	42	22	42	− 2.2	2.0E–04
303*°	54,773 (8.9)	SwissProt	FUMH_HUMAN	FH	Fumarate hydratase, mitochondrial	583	38	24	41	− 2.1	1.7E–05
342*	46,915 (6.5)	SwissProt	IDHC_HUMAN	IDH1	Isocitrate dehydrogenase [NADP] cytoplasmic	940	53	25	45	− 2.0	9.0E–04
416*	38,837 (9.2)	SwissProt	ROA1_HUMAN	HNRNPA1	Heterogeneous nuclear ribonucleoprotein A	478	34	16	37	− 2.0	4.4E–04
	35,937 (9.0)	SwissProt	MDHM_HUMAN	MDH2	Malate dehydrogenase, mitochondrial	383	28	17	42	–	–
247*°	59,828 (9.2)	SwissProt	ATPA_HUMAN	ATP5F1A	ATP synthase subunit alpha, mitochondrial	3866	218	32	42	− 2.0	5.3E–05
259*°	55,454 (6.3)	SwissProt	AL1A1_HUMAN	ALDH1A1	Retinal dehydrogenase I	2546	184	29	48	− 1.9	3.9E–05
864	28,061 (5.3)	NCBInr	NP_000030.1	APOA1	Apolipoprotein A-I isoform 1 preproprotein	707	102	26	74	− 1.8	9.3E–05
199*	59,947 (6.9)	SwissProt	CATA_HUMAN	CAT	Catalase	612	41	27	37	− 1.8	1.1E–03
305*°	44,101 (6.1)	SwissProt	PA2G4_HUMAN	PA2G4	Proliferation-associated protein 2G4	488	32	20	45	− 1.7	5.1E–03
517*	26,325 (8.5)	NCBInr	gi|119,615,235	SDHB	Succinate dehydrogenase complex, subunit B, iron sulfur (Ip), isoform CRA_b	261	31	15	47	− 1.7	2.0E–05
253°	52,908 (7.1)	NCBInr	gi|237,823,915	FGB	Chain B, crystal structure off human fibrinogen	894	35	20	45	− 1.7	9.6E–04
268	59,828 (9.2)	SwissProt	ATPA_HUMAN	ATP5F1A	ATP synthase subunit alpha, mitochondrial	824	59	29	41	− 1.7	1.0E–03
	56,315 (6.7)	NCBInr	gi|20,151,189	GLUD1	Chain A, Structure of human glutamate dehydrogenase-apo form	565	51	26	44	–	–
261*°	56,525 (5.3)	SwissProt	ATPB_HUMAN	ATP5F1B	ATP synthase subunit beta, mitochondrial	6610	353	23	50	− 1.6	9.6E–03
336*	49,852 (7.3)	SwissProt	EFTU_HUMAN	TUFM	Elongation factor Tu, mitochondrial	827	58	22	46	− 1.6	4.4E–03
106*°	73,920 (5.9)	SwissProt	GRP75_HUMAN	HSPA9	Stress-70 protein, mitochondrial	1042	55	34	43	− 1.6	4.9E–04
450*	42,902 (5.3)	SwissProt	KCRB_HUMAN	CKB	Creatine kinase B-type	1627	79	59	49	− 1.6	4.9E–03
262	59,828 (9.2)	SwissProt	ATPA_HUMAN	ATP5F1A	ATP synthase subunit alpha, mitochondrial	1864	123	25	43	− 1.6	3.8E–03
257*	55,454 (10.9)	SwissProt	AL1A1_HUMAN	ALDH1A1	Retinal dehydrogenase 1	1093	62	24	41	− 1.6	5.9E–03
252*°	54,143 (8.0)	SwissProt	DLDH_HUMAN	DLD	Dihydrolipoyl dehydrogenase, mitochondrial	299	29	17	21	− 1.6	4.7E–04
162*°	67,144 (9.0)	SwissProt	AIFM1_HUMAN	AIFM1	Apoptosis-inducing factor 1, mitochondrial	784	33	20	30	− 1.6	2.1E–04
111*°	72,402 (5.1)	SwissProt	GRP78_HUMAN	HSPA5	78 kDa glucose-regulated protein (endoplasmic reticulum chaperone BiP)	10,489	469	52	62	− 1.5	4.1E–05

Proteins are sorted by fold change

^a^Spot numbers refer to Fig. 1. Five spots (11, 57, 268, 416, and 865) contained two proteins

^b^Molecular weight in Daltons

^c^Called “average ratio” by DeCyder

^d^Also ERP57

^*^Spots also differential in GC vs. controls

°Spots also differential in FDR-GC vs. control

According to DAVID Bioinformatics Resources, proteins more abundant in AAG-corpus were involved (adjusted *P* < 0.05) in the molecular functions “structural molecule activity” (GO: 0005198; including *FGA*, *KRT19*, *KRT18*, *LMNA*, *VIM*), “cadherin binding involved in cell–cell adhesion” (GO:0098641; including *ANXA2, HSPA1A, KRT18*, *PRDX1*, *YWHAZ*) (DAVID_raw data, sheet 1), and “protein binding” (GO:0005515; including *CA1*, *CFL1*, *VIM*, *ARHGDIB*, *PRXD1*). Regarding biological processes, proteins less abundant in AAG-corpus than controls were involved in “tricarboxylic acid cycle” (GO:0006099; including *ACO2*, *DLD*, *FH*, *IDH1*, *IDH2*, *MDH2*, *SDHB*), followed by “isocitrate metabolic process” (GO:0006102; including *ACO2*, *IDH1*, *IDH2*) and “malate metabolic process” (GO:0006108; including *FH*, *LIPF*, *MDH2*) (DAVID_raw data, sheet 2).

### Protein abundance in AAG vs. other gastric conditions

In Part II, we examined the levels of the 67 spots (53 differentially abundant proteins) in four other sets of specimens (Table 1). Two sets were gastric antrum biopsies from AAG patients without and with confirmed *H. pylori* infection (called AAG-antrum and AAG-antrum-HP, respectively); two out of three patients in each group had intestinal metaplasia. Two additional sets were gastric biopsies from first-degree relatives of GC patients (FDR-GC) and from GC patients.

**Fig. 3:**
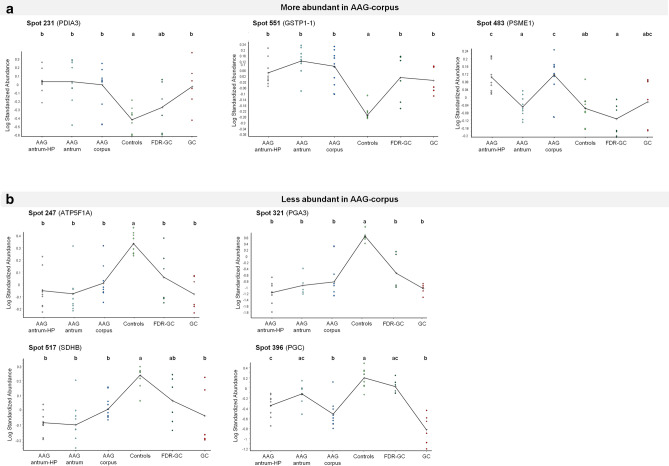
Abundances of seven differentially abundant spots in the five experimental groups and controls, expressed as log standardized abundance from 2D-DIGE. Dots mark individual samples, and the line connects group mean values. Different letters indicate significantly different groups (*P* < 0.01, one-way ANOVA).** a** Three selected spots more abundant in AAG-corpus than controls;** b** Four selected spots less abundant in AAG-corpus than controls. Data for the remaining 60 spots are shown in Figure S1

Examination of proteome maps from the AAG-antrum and AAG-antrum-HP groups revealed that, of the 67 spots identified in Part I, 57 were also differentially abundant (vs. controls) in these groups (*P* < 0.01) with the same directions of effect, and one spot (548) was differentially abundant but with the opposite direction. The 57 concordant spots corresponded to 43 unique proteins. Of the remaining nine spots, two (127 and 415) had similar levels as controls, two (111 and 305) were differentially abundant only in AAG-antrum, and five (131, 203, 232, 483 and 783) were differentially abundant only in AAG-antrum-HP. Log_10_ standardized abundance values for seven spots are shown in Fig. 3, and for the remaining 60 spots in Fig. S1.

Examination of GC proteome maps revealed that 14 of the 28 spots more abundant in AAG-corpus (than controls) were also more abundant in GC (see asterisks in Table 2). Furthermore, 35 of the 39 spots less abundant in AAG-corpus (than controls) were also less abundant in GC. Altogether, 30 unique proteins that were differentially abundant in AAG-corpus were also differentially abundant in GC. Examination of FDR-GC proteome maps revealed that 6 of the 28 spots more abundant in AAG-corpus were also more abundant in FDR-GC (see circles in Table 2), and 25 of the 39 spots less abundant were also less abundant in FDR-GC, for a total of 23 unique proteins maintaining their differential abundance status.

### Validation of differentially abundant proteins

In Part III, seven spots (seven distinct proteins) identified in Part I were chosen for validation (Fig. 3). These spots included three that were more abundant (Fig. 3a) and four that were less abundant (Fig. 3b) in AAG-corpus than controls. Two spots (231 and 551) were selected because their abundance was significantly higher in all AAG groups and GC than in controls. The spots were identified as chain A, protein disulfide-isomerase A3 erp57 (*PDIA3* or *ERP57*; spot 231), and glutathione S-transferase P1-1 class (*GSTP*; spot 551); this latter spot was also more abundant in FDR-GC. Spot 483 was more abundant in AAG-corpus and AAG-antrum-HP than in controls, while its abundance in GC was similar to that of controls; this spot was identified as proteasome activator complex subunit 1 (*PSME1*). Three spots (247, 321 and 517) were selected because their abundance in all AAG groups and GC was significantly lower than in controls. Spots 247 and 321 were also significantly less abundant in FDR-GC, while spot 517 had similar levels between FDR-GC and controls. These three spots were identified as ATP synthase subunit alpha, mitochondrial (*ATP5F1A*; spot 247), pepsinogen-3 (*PGA3*; spot 321), and succinate dehydrogenase complex, subunit B, iron sulfur (Ip), isoform CRA_b (*SDHB*; spot 517). Finally, spot 396, identified as pepsinogen C (*PGC*), was selected for being more abundant in AAG-antrum than AAG-antrum-HP, AAG-corpus and GC, in accordance with the physiological production of pepsinogen C by the antrum. Spots 321 (pepsinogen-3) and 396 (pepsinogen C) were also selected for their association with gastric diseases.

**Fig. 4:**
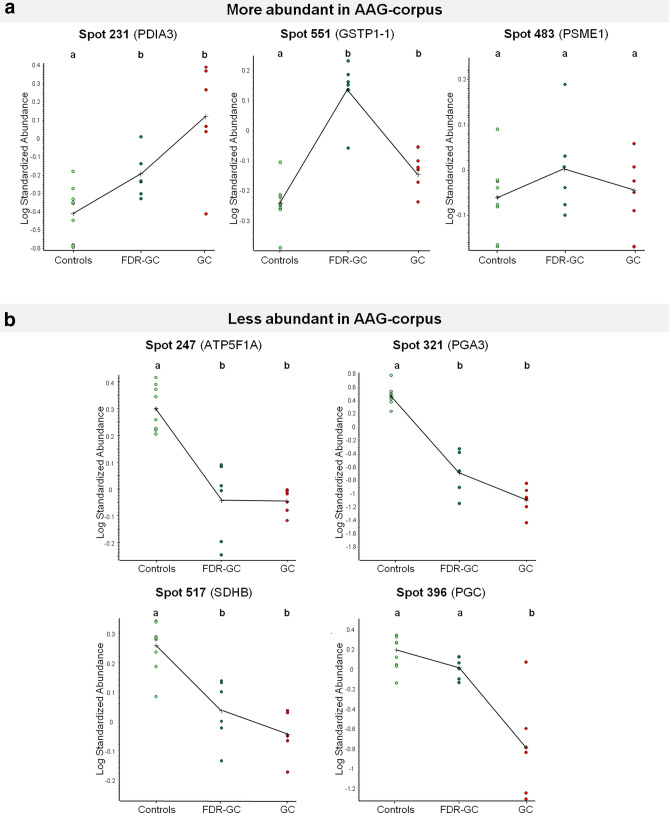
Validation analysis for seven differentially abundant spots in two additional sets of gastric biopsies vs. controls, expressed as log standardized abundance from 2D-DIGE. Dots mark individual samples, and the line connects group mean values. Different letters indicate significantly different groups (*P* < 0.01, one-way ANOVA).** a** Three selected spots more abundant in AAG-corpus than controls;** b** Four selected spots less abundant in AAG-corpus than controls

First, 2D-DIGE was repeated in an attempt to validate the findings for the seven spots in additional FDR-GC and GC biopsy samples (Part III in Table 1). For the three spots more abundant in AAG-corpus (Fig. 4a), this analysis confirmed the higher abundance (vs. controls) of PDIA3 in GC, the higher abundance GSTP1-1 in both FDR-GC and GC, and the similar levels to controls for PSME1 in both groups. For the four spots less abundant in AAG-corpus (Fig. 4b), this analysis confirmed the lower abundance of all in GC, while for FDR-GC it confirmed the lower levels of ATP5F1A and PGA3 and the similar abundance of PGC.

**Fig. 5:**
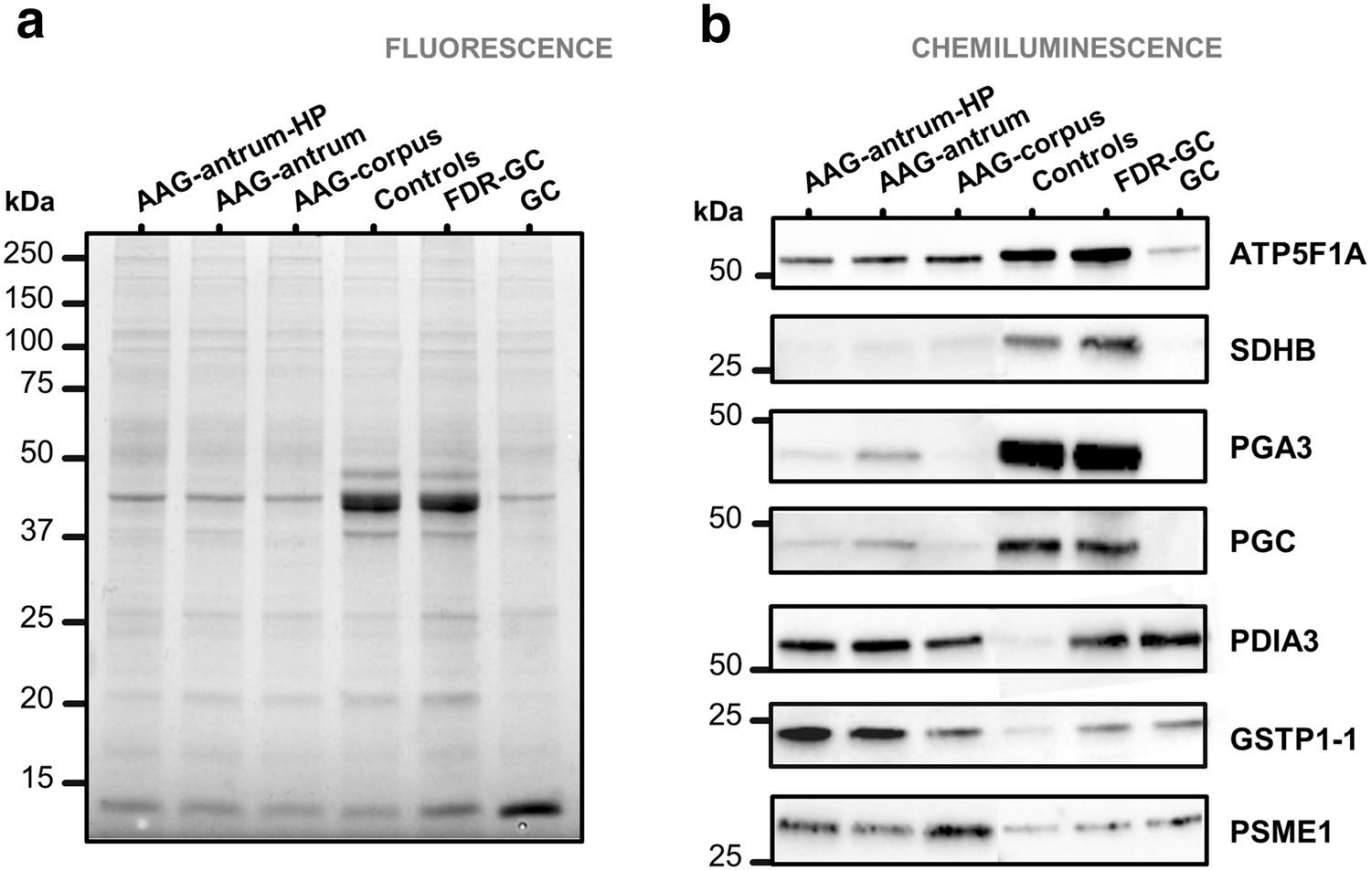
Immunochemical detection of seven differentially abundant proteins, indicated by gene names on the right. Samples are pools of extracted protein from six gastric biopsy specimens per study group.** a** Image of the gel acquired with Chemidoc system before transfer to nitrocellulose membranes.** b** Western blots of the selected proteins

Finally, the abundance of the seven selected proteins was examined by immunoblotting of pooled proteins (Fig. 5). This analysis confirmed the lower levels of ATP synthase subunit alpha (*ATP5F1A*), succinate dehydrogenase complex subunit B (*SDHB*), pepsinogen-3 (*PGA3*), and pepsinogen C (*PGC*) in all three AAG groups and in GC than in controls. In FDR-GC, immunoblotting confirmed the similar abundances of PGC and PSME1, and the higher abundance of GSTP1-1, but did not confirm the similar abundance of SDHB and PDIA3 or the lower abundance of ATP5F1A or PGA3. The analysis also confirmed the higher levels of PDIA3 in AAG and GC than in controls. For GSTP1-1, immunoblotting confirmed the higher abundance in all AAG groups and in GC than in controls. Finally, for PSME1, the higher abundance in AAG-antrum-HP and AAG-corpus than in controls was confirmed.

## Discussion

This study identified a gastric tissue profile comprising 53 corpus proteins differentially abundant between AAG-corpus and controls. Of these proteins, 25 were more abundant and 28 were less abundant in AAG-corpus. These results were also obtained for 43 proteins in AAG-antrum biopsies, irrespective of *H. pylori* infection status. In GC biopsies and gastric biopsies of first-degree relatives of GC patients, 30 and 23 proteins, respectively, maintained their differential abundance status. At the individual protein level, the gastric tissue profile includes PDIA3, GSTP1-1, and PSME1, which were more abundant in AAG-corpus, and ATP5F1A, PGA3, PGC,, and SDHB, which were less abundant. These proteins were also differentially abundant in AAG-antrum, AAG-antrum-HP, and GC. Regarding FDR-GC, only PDIA3 and GSTP1-1 maintained the differential status.

The proteomics profile of AAG-corpus was partially observed in biopsies of the antrum (for 43 of 53 unique proteins), reflecting a common response of the gastric mucosa to chronic inflammation. However, ten proteins did not have the same changes in abundance, and some differences were found between antrum biopsies with and without *H. pylori* infection. The lack of complete overlap may reflect real pathophysiological differences, or may be an artifact due to small sample number and is worthy of further investigation.

According to DAVID, the 28 less abundant proteins were enriched in “tricarboxylic acid (TCA) cycle”, suggesting that the gastric corpus in AAG has TCA cycle impairments. The decreased abundance of proteins of the TCA cycle agrees with previous findings of decreased respiratory capacity and deficient mitochondrial respiratory complex I in corpus mucosal cells of patients with atrophic gastritis [32]. It is possible that, in AAG, loss of zymogenic mucosal cells and consequent reduction of zymogen granule secretion reduce energy demand. Moreover, energy metabolism (i.e. TCA cycle and oxidative phosphorylation) may be decreased by lower iron availability [33] due to achlorhydria [34].

We found that proteins involved in “structural molecule activity” and “cadherin binding involved in cell–cell adhesion” were more abundant in AAG tissues. In AAG, autoantibodies against parietal cells cause apoptosis and atrophy in the corpus mucosa [35]. An increased synthesis of intercellular adhesion molecules by oxyntic mucosa cells may reflect an attempt to counteract apoptosis and atrophy. Thus, the observed increase in abundance of some proteins with adhesive properties in AAG may be a reaction of the atrophic gastric mucosa to parietal cell loss and gastric cell phenotype changes.

This study has several limitations. First, the sample size is small and does not reflect the range of severity of AAG, from pre-atrophic to severe atrophic. In all AAG groups, two out of three patients had advanced atrophy with intestinal metaplasia, but how this condition affected the proteomics profiles could not be investigated. Intestinal metaplasia is a common finding in AAG; hence we included these patients as they are part of the spectrum of AAG histopathology and natural history. Another limitation is the heterogeneity of GC samples (intestinal, diffuse and indeterminate), which precluded identifying differences related to histology but made our protein signature more reflective of all GC types.

### Possible roles of selected differentially abundant proteins

Protein disulfide isomerase A3 (PDIA3) was more abundant in all AAG groups and in GC than controls. It is a member of the protein disulfide isomerase-like family [36] and an endoplasmic reticulum chaperone implicated in oncogenesis, tumor progression, and immune response of carcinoma cells [37–39]. PDIA3 has been found increased in spasmolytic polypeptide-expressing metaplasia, a reparative tissue that develops in response to oxyntic atrophy [40] and *H. pylori* infection [41]. High levels of PDIA3 in gastric adenocarcinoma have been associated with better prognosis [37, 42].

GST protein P1-1 class (GSTP1-1) was also more abundant in all AAG groups and GC than controls. It belongs to the glutathione S-transferase family, which has various functions (e.g., detoxification of exogenous substances) [43, 44]. The increased abundance of GSTP1-1 in AAG may be a mechanism to counteract oxidative stress, which is known to occur in stomach disorders, including gastritis [45].

Proteasome activator complex subunit 1 (PSME1) was more abundant in AAG-corpus and AAG-antrum-HP than controls. It is a multicatalytic proteinase complex, involved in immunoproteasome assembly required for efficient antigen processing [46]. Its abundance in AAG-antrum-HP suggests a protective function against *H. pylori* infection. PSME1 content in GC was similar to that in controls.

ATP synthase subunit alpha (ATP5F1A) was less abundant in all AAG groups and GC than controls. It is a mitochondrial protein that generates ATP in the presence of an H^+^ gradient across membranes [47]. Reduced ATP synthase abundance in AAG-corpus may reflect a decreased energy demand by gastric tissues, consistent with the above-described TCA cycle impairment and lower SDHB abundance. Decreased ATP synthase in AAG-antrum, irrespective of *H. pylori* infection, and in GC may reflect reduced energy demand and respiratory activity in these diseases.

Pepsinogen 3 (PGA3) abundance was also less abundant in all AAG groups and GC than controls. It is an aspartic protease primarily secreted by gastric chief cells (zymogenic), and it is activated into the digestive enzyme pepsin when it comes in contact with acid produced by parietal cells. Aspartic proteinases are classified into two major groups: pepsinogen I or A, to which PGA3 belongs, and pepsinogen II or C [48]. Low pepsinogen content may reflect the loss of parietal cells. Our results confirm those of Kuipers et al. [49], who found at the gene level a loss of PGA3 expression in patients with atrophic gastritis or GC.

Pepsinogen C (PGC) was less abundant in AAG-corpus, AAG-antrum-HP, and GC than controls. These results agree with a previous study that showed progressively lower PGC expression, compared to normal gastric mucosa, in lesions of increasing severity, namely gastric erosions, atrophic gastritis, and GC (where only 2.4% of specimens had detectable levels) [50].

Succinate dehydrogenase complex, subunit B (SDHB) was also less abundant in all AAG groups and GC than controls. It is involved in the mitochondrial electron transport chain (complex II), a subpathway of the TCA cycle and is part of carbohydrate metabolism [51]. Decreased abundance of both SDHB and ATP5F1A in GC was recently reported [52].

## Conclusion

This study identified a proteomics signature of stomach corpus in AAG, which includes decreased abundance of proteins involved in the TCA cycle and increased abundance of those in structural molecule activity and cadherin binding. Many of these AAG markers are shared with GC. These proteomics alterations may represent a link between AAG and GC and be part of the progression to gastric cancerogenesis. Our proteomic approach on tissue should be integrated with transcriptomic and biochemical data.

## Supplementary Information

Below is the link to the electronic supplementary material.

Supplementary file1 (PDF 780 KB)

Supplementary file2 (XLS 64 KB)

## References

[1] Lenti MV, Rugge M, Lahner E, Miceli E, Toh BH, Genta RM (2020). Autoimmune gastritis. Nat Rev Dis Primers.

[2] Di Sabatino A, Lenti MV, Giuffrida P, Vanoli A, Corazza GR (2015). New insights into immune mechanisms underlying autoimmune diseases of the gastrointestinal tract. Autoimmun Rev.

[3] Mårdh S, Song YH (1989). Characterization of antigenic structures in auto-immune atrophic gastritis with pernicious anaemia. The parietal cell H, K-ATPase and the chief cell pepsinogen are the two major antigens. Acta Physiol Scand..

[4] Lahner E, Norman GL, Severi C, Encabo S, Shums Z, Vannella L (2009). Reassessment of intrinsic factor and parietal cell autoantibodies in atrophic gastritis with respect to cobalamin deficiency. Am J Gastroenterol.

[5] Chlumska A, Boudova L, Benes Z, Zámecník M (2005). Autoimmune gastritis. A clinicopathologic study of 25 cases. Cesk Patol..

[6] Solcia E, Fiocca R, Villani L, Luinetti O, Capella C (1995). Hyperplastic, dysplastic, and neoplastic enterochromaffin-like-cell proliferations of the gastric mucosa. Classification and histogenesis. Am J Surg Pathol..

[7] Carmel R (1988). Pepsinogens and other serum markers in pernicious anemia. Am J Clin Pathol.

[8] Rusak E, Chobot A, Krzywicka A, Wenzlau J (2016). Anti-parietal cell antibodies—diagnostic significance. Adv Med Sci.

[9] Khan S, Del-Duca C, Fenton E, Holding S, Hirst J, Doré PC (2009). Limited value of testing for intrinsic factor antibodies with negative gastric parietal cell antibodies in pernicious anaemia. J Clin Pathol.

[10] Magris R, De Re V, Maiero S, Fornasarig M, Guarnieri G, Caggiari L (2020). Low pepsinogen I/II ratio and high gastrin-17 levels typify chronic atrophic autoimmune gastritis patients with gastric neuroendocrine tumors. Clin Transl Gastroenterol.

[11] Miceli E, Padula D, Lenti MV, Gallia A, Albertini R, Di Stefano M (2015). A laboratory score in the diagnosis of autoimmune atrophic gastritis: a prospective study. J Clin Gastroenterol.

[12] Strickland RG, Van der Reis L (1975). Gastritis. Immune disorders. Front Gastrointest Res.

[13] Strickland RG, Mackay IR (1973). A reappraisal of the nature and significance of chronic atrophic gastritis. Am J Dig Dis.

[14] Veijola LI, Oksanen AM, Sipponen PI, Rautelin HI (2010). Association of autoimmune type atrophic corpus gastritis with *Helicobacter pylori* infection. World J Gastroenterol.

[15] Miceli E, Vanoli A, Lenti MV, Klersy C, Di Stefano M, Luinetti O (2019). Natural history of autoimmune atrophic gastritis: a prospective, single centre, long-term experience. Aliment Pharmacol Ther.

[16] Bizzaro N, Antico A, Villalta D (2018). Autoimmunity and Gastric Cancer. Autoimmune gastritis has been associated with the development of two types of gastric neoplasms: intestinal type and type I gastric carcinoid. Int J Mol Sci..

[17] Lahner E, Esposito G, Galli G, Annibale B (2015). Atrophic gastritis and pre-malignant gastric lesions. Transl Gastrointest Cancer.

[18] Kokkola A, Sjoblom SM, Haapiainen R, Sipponen P, Puolakkainen P, Järvinen H (1998). The risk of gastric carcinoma and carcinoid tumours in patients with pernicious anaemia. A prospective follow-up study. Scand J Gastroenterol..

[19] Kodama M, Murakami K, Okimoto T, Abe H, Sato R, Ogawa R (2013). Histological characteristics of gastric mucosa prior to *Helicobacter pylori* eradication may predict gastric cancer. Scand J Gastroenterol.

[20] Vannella L, Lahner E, Osborn J, Bordi C, Miglione M, Delle Fave G (2010). Risk factors for progression to gastric neoplastic lesions in patients with atrophic gastritis. Aliment Pharmacol Ther.

[21] Minalyan A, Benhammou JN, Artashesyan A, Lewis MS, Pisegna JR (2017). Autoimmune atrophic gastritis: current perspectives. Clin Exp Gastroenterol.

[22] Toh BH (2014). Diagnosis and classification of autoimmune gastritis. Autoimmun Rev.

[23] Kondo T (2019). Cancer biomarker development and two-dimensional difference gel electrophoresis (2D-DIGE). Biochim Biophys Acta Proteins Proteom.

[24] Wu JY, Cheng CC, Wang JY, Wu DC, Hsieh JS, Lee SC (2014). Discovery of tumor markers for gastric cancer by proteomics. PLoS ONE.

[25] Zhang J, Song MQ, Zhu JS, Zhou Z, Xu ZP, Chen WX (2011). Identification of differentially-expressed proteins between early submucosal non-invasive and invasive colorectal cancer using 2D-DIGE and mass spectrometry. Int J Immunopathol Pharmacol.

[26] Moriggi M, Pastorelli L, Torretta E, Tontini GE, Capitanio D, Bogetto SF, et al. Contribution of extracellular matrix and signal mechanotransduction to epithelial cell damage in inflammatory bowel disease patients: a proteomic study. Proteomics. 2017;17. 10.1002/pmic.201700164 29027377

[27] Song M, Camargo MC, Weinstein SJ, Best AF, Männistö S, Albanes D, Rabkin CS (2018). Family history of cancer in first-degree relatives and risk of gastric cancer and its precursors in a Western population. Gastric Cancer.

[28] Dixon MF, Genta RM, Yardley JH, Correa P (1996). Classification and grading of gastritis. The updated Sydney System. International Workshop on the Histopathology of Gastritis, Houston 1994. Am J Surg Pathol.

[29] Lauren P (1965). The two histologic main types of gastric carcinoma: diffuse and so-called intestinal type carcinoma. An attempt at a histo-clinical classification. Acta Parhol Microbid Scan..

[30] Di Sabatino A, Biagi F, Lenzi M, Frulloni L, Lenti MV, Giuffrida P (2017). Clinical usefulness of serum antibodies as biomarkers of gastrointestinal and liver diseases. Dig Liver Dis.

[31] da Huang W, Sherman BT, Lempicki RA (2009). Systematic and integrative analysis of large gene lists using DAVID bioinformatics resources. Nat Protoc.

[32] Gruno M, Peet N, Tein A, Salupere R, Sirotkina M, Valle J (2008). Atrophic gastritis: deficient complex I of the respiratory chain in the mitochondria of corpus mucosal cells. J Gastroenterol.

[33] Oexle H, Gnaiger E, Weiss G (1999). Iron-dependent changes in cellular energy metabolism: influence on citric acid cycle and oxidative phosphorylation. Biochim Biophys Acta.

[34] Betesh AL, Santa Ana CA, Cole JA, Fordtran JS (2015). Is achlorhydria a cause of iron deficiency anemia?. Am J Clin Nutr.

[35] Bergman MP, Vandenbroucke-Grauls CM, Appelmelk BJ, D'Elios MM, Amedei A, Azzurri A (2005). The story so far: *Helicobacter pylori* and gastric autoimmunity. Int Rev Immunol.

[36] Zhang Y, Baig E, Williams DB (2006). Functions of ERp57 in the folding and assembly of major histocompatibility complex class I molecules. J Biol Chem.

[37] Shimoda T, Wada R, Kure S, Ishino K, Kudo M, Ohashi R (2019). Expression of protein disulfide isomerase A3 and its clinicopathological association in gastric cancer. Oncol Rep.

[38] Coe H, Michalak M (2010). ERp57, a multifunctional endoplasmic reticulum resident oxidoreductase. Int J Biochem Cell Biol.

[39] Cicchillitti L, Di Michele M, Urbani A, Ferlini C, Donat MB, Scambia G (2009). Comparative proteomic analysis of paclitaxel sensitive A2780 epithelial ovarian cancer cell line and its resistant counterpart A2780TC1 by 2D-DIGE: the role of ERp57. J Proteome Res.

[40] Nomura S, Yamaguchi H, Ogawa M, Wang TC, Lee JR, Goldenring JR (2005). Alterations in gastric mucosal lineages induced by acute oxyntic atrophy in wild-type and gastrin-deficient mice. Am J Physiol Gastrointest Liver Physiol.

[41] Lee JR, Baxter TM, Yamaguchi H, Wang TC, Goldenring JR, Anderson MG (2003). Differential protein analysis of spasomolytic polypeptide expressing metaplasia using laser capture microdissection and two-dimensional difference gel electrophoresis. Appl Immunohistochem Mol Morphol.

[42] Leys CM, Nomura S, LaFleur BJ, Ferrone S, Kaminishi M, Montgomery E (2007). Expression and prognostic significance of prothymosin-alpha and ERp57 in human gastric cancer. Surgery.

[43] Singh S (2015). Cytoprotective and regulatory functions of glutathione S-transferases in cancer cell proliferation and cell death. Cancer Chemoth Pharm.

[44] Hayes JD, Flanagan JU, Jowsey IR (2005). Glutathione transferases. Annu Rev Pharmacol Toxicol.

[45] Suzuki H, Nishizawa T, Tsugawa H, Mogami S, Hibi T (2012). Roles of oxidative stress in stomach disorders. J Clin Biochem Nutr.

[46] Vigneron N, van den Eynde BJ (2014). Proteasome subtypes and regulators in the processing of antigenic peptides presented by class I molecules of the major histocompatibility complex. Biomolecules.

[47] Junge W, Nelson N (2015). ATP synthase. Annu Rev Biochem.

[48] Gritti I, Banfi G, Roi GS (2000). Pepsinogens: physiology, pharmacology pathophysiology and exercise. Pharmacol Res.

[49] Kuipers EJ, Peña AS, Crusius JB, Defize J, van der Stoop P, Meuwissen SG, Pals G (1995). Absence of pepsinogen A3 gene expression in the gastric mucosa of patients with gastric cancer. J Clin Pathol.

[50] Ning PF, Liu HJ, Yuan Y (2005). Dynamic expression of pepsinogen C in gastric cancer, precancerous lesions and *Helicobacter pylori* associated gastric diseases. World J Gastroenterol.

[51] Rutter J, Winge DR, Schiffman JD (2010). Succinate dehydrogenase—assembly, regulation and role in human disease. Mitochondrion.

[52] Fernández-Coto DL, Gil J, Hernández A, Herrera-Goepfert R, Castro-Romero I, Hernández-Márquez E (2018). Quantitative proteomics reveals proteins involved in the progression from non-cancerous lesions to gastric cancer. J Proteomics.

