# Supplementary figures and images for: Proteomics signature of autoimmune atrophic gastritis: towards a link with gastric cancer
Source: Gastric Cancer. 2021 Feb 23;24(3):666–79. doi: 10.1007/s10120-020-01148-3 (PMC8064991; doi:10.1007/s10120-020-01148-3)

**(A)** More abundant in AAG-corpus

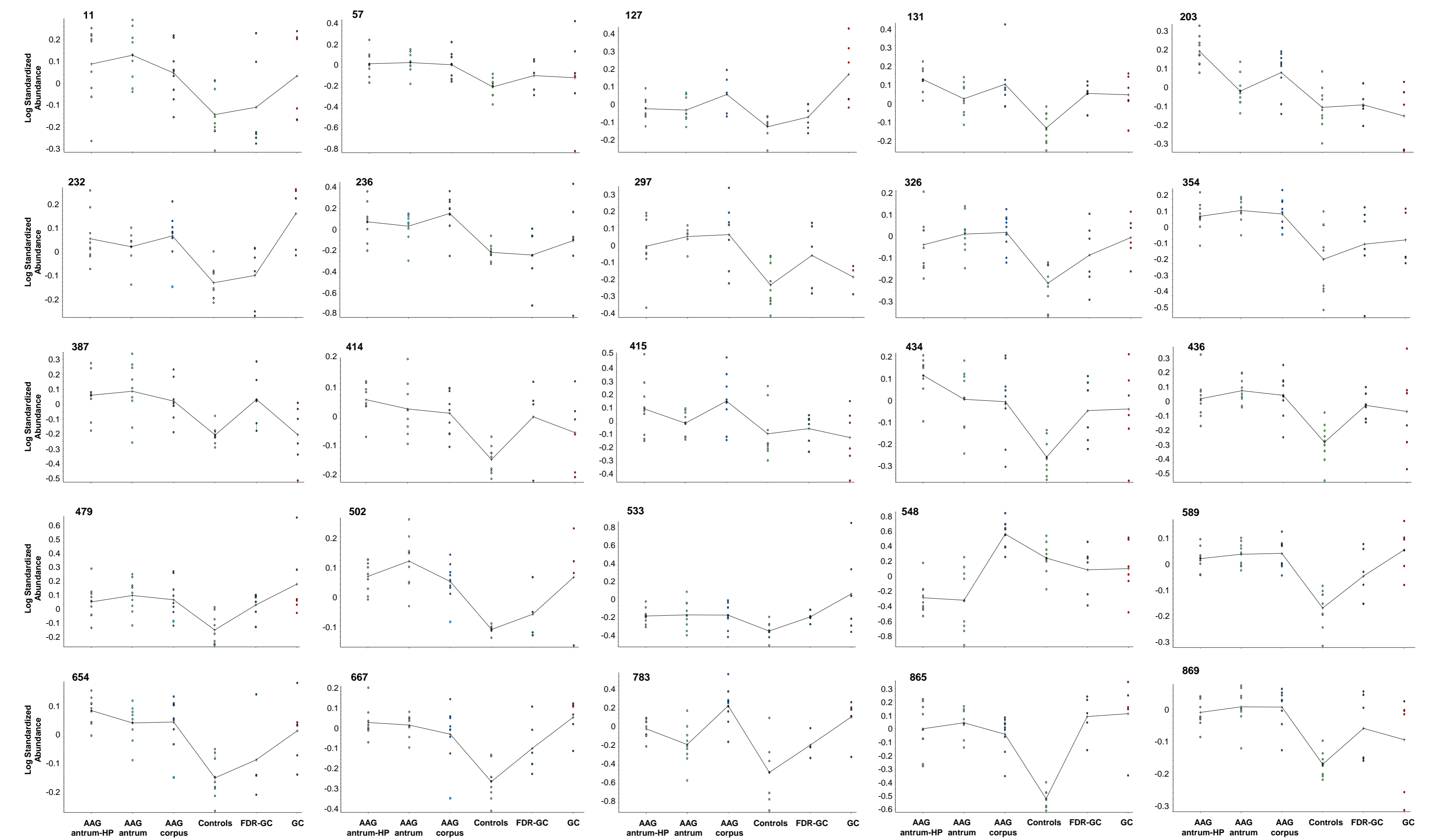

(B)

Less abundant in AAG-corpus

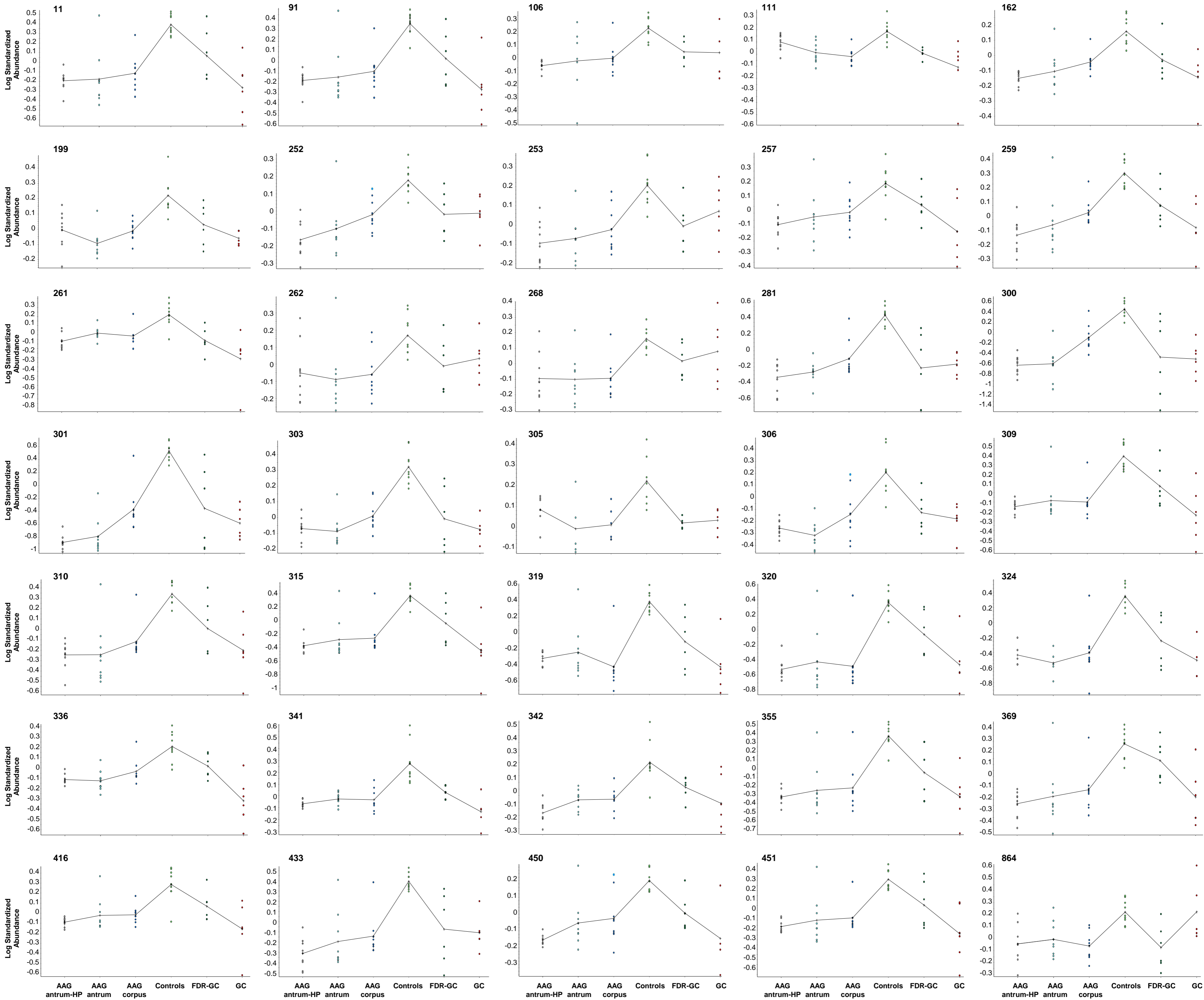

Supplement: Supplementary file 1 — Supplementary file1 (PDF 780 KB) [file 10120_2020_1148_MOESM1_ESM.pdf]
